# Immune Biomarkers for Diagnosis and Treatment Monitoring of Tuberculosis: Current Developments and Future Prospects

**DOI:** 10.3389/fmicb.2019.02789

**Published:** 2019-12-18

**Authors:** Yean K. Yong, Hong Y. Tan, Alireza Saeidi, Won F. Wong, Ramachandran Vignesh, Vijayakumar Velu, Rajaraman Eri, Marie Larsson, Esaki M. Shankar

**Affiliations:** ^1^Laboratory Center, Xiamen University Malaysia, Sepang, Malaysia; ^2^Department of Traditional Chinese Medicine, Xiamen University Malaysia, Sepang, Malaysia; ^3^Department of Pediatrics, Emory Vaccine Center, Atlanta, GA, United States; ^4^Department of Medical Microbiology, Faculty of Medicine, University of Malaya, Kuala Lumpur, Malaysia; ^5^Department of Microbiology and Immunology, Emory Vaccine Center, Atlanta, GA, United States; ^6^School of Health Sciences, College of Health and Medicine, University of Tasmania, Launceston, TAS, Australia; ^7^Division of Molecular Virology, Department of Clinical and Experimental Medicine, Linkoping University, Linkoping, Sweden; ^8^Division of Infection Biology and Medical Microbiology, Department of Life Sciences, Central University of Tamil Nadu (CUTN), Thiruvarur, India

**Keywords:** biomarkers, diagnostics, treatment monitoring, tuberculosis, HIV

## Abstract

Tuberculosis (TB) treatment monitoring is paramount to clinical decision-making and the host biomarkers appears to play a significant role. The currently available diagnostic technology for TB detection is inadequate. Although GeneXpert detects total DNA present in the sample regardless live or dead bacilli present in clinical samples, all the commercial tests available thus far have low sensitivity. Humoral responses against *Mycobacterium tuberculosis* (Mtb) antigens are generally low, which precludes the use of serological tests for TB diagnosis, prognosis, and treatment monitoring. Mtb-specific CD4^+^ T cells correlate with Mtb antigen/bacilli burden and hence might serve as good biomarkers for monitoring treatment progress. Omics-based techniques are capable of providing a more holistic picture for disease mechanisms and are more accurate in predicting TB disease outcomes. The current review aims to discuss some of the recent advances on TB biomarkers, particularly host biomarkers that have the potential to diagnose and differentiate active TB and LTBI as well as their use in disease prognosis and treatment monitoring.

## Introduction

Tuberculosis (TB) represents a major public health problem worldwide, and is transmitted via inhalation of aerosolized droplets carrying *Mycobacterium tuberculosis* (Mtb). It is estimated that approximately one-third (∼2 billion) of the global population is living with latent TB (LTBI) (World Health Organization [WHO], 2002), of which ∼10% likely progress to develop active TB within 2 years after initial exposure to the tubercle bacilli (Corbett et al., 2003). The risk of reactivation of latent TB is remarkably high among individuals infected with the human immunodeficiency virus (HIV) as well as in individuals on long-term immunosuppressive treatment with TNF-α inhibitors (Selwyn et al., 1989; Getahun et al., 2010; Day et al., 2018; Amelio et al., 2019). According to the latest WHO Global TB Report, Mtb has led to 10 million cases of TB in 2017, and is also one of the top 10 causes of mortality with ∼1.6 million (1.3 and 0.3 million in HIV-negative and HIV-positive individuals, respectively) deaths in 2017, which translates to ∼4000 deaths each day (Tiberi et al., 2018; World Health Organization [WHO], 2018). Although universal Bacillus Calmette-Guérin (BCG) is administered in many countries, the vaccine is only effective against disseminated TB in children, and it’s efficacy in adults largely remains controversial (Colditz et al., 1994; Zwerling et al., 2011; Amelio et al., 2017).

Tuberculosis treatment monitoring is paramount to clinical decision-making and the host biomarkers appears to play a significant role. Patients diagnosed with TB are generally put under a four drugs regimen (isoniazid, rifampicin, pyrazinamide, and ethambutol) for 2 months, known as the intensive phase; followed by 4 months maintenance phase with isoniazid and rifampicin. In spite of 6-long-months of anti-TB therapy, some patients however, will experience recurrence of infection and have an increased risk of M/XDR-TB. This 6-month duration may lead to prohibitive delay for clinical management. Exposure of Mtb to suboptimal drug concentrations risks robust bacterial replication and dissemination, increased rates of transmission and development of drug resistance. WHO reported an estimated 558,000 cases of rifampicin-resistant TB in 2017, of which 82% are infected with MDR-TB (World Health Organization [WHO], 2018). This has caused significant complications to the patients as they are to be treated with second-line drugs for an even longer duration (18–24 months) (World Health Organization [WHO], 2016), despite that their survival rate was merely <50% (World Health Organization [WHO], 2017). Hence, biomarkers that indicate an efficacious treatment at the early therapeutic phase as well as at the end of treatment, which predicts relapse could enormously improve clinical prognosis.

In order to achieve global control of TB disease, development of an effective novel vaccine (Zwerling et al., 2011) and novel drugs with shortened treatment duration (Abu-Raddad et al., 2009; Argun et al., 2016; Tiberi et al., 2018), as well as simpler and more accurate diagnostic tests (Walzl et al., 2011, 2018; Goletti et al., 2016, 2018) are of upmost importance. Hence, there is a pressing need to develop a low cost, minimal-invasive, non-sputum-based, highly sensitive and specific TB diagnostic test that uses easily accessible biological specimens such as blood and urine (Strimbu and Tavel, 2010; Nalejska et al., 2014; World Health Organization [WHO], 2014; Ballman, 2015; Buschmann et al., 2016; Goletti et al., 2016, 2018). Biomarkers can generally be divided into: (i) Mtb components, (ii) antibody responses to Mtb antigens, (iii) cellular immune responses to Mtb antigens, and (iv) unbiased “omics” approach (i.e., transcriptomics, proteomics and metabolomics). Here, we discussed some of the recent advances on TB biomarkers, particularly host biomarkers that have the potential to diagnose and differentiate active TB and LTBI as well as their use in disease prognosis and treatment monitoring. References for this review were identified through searches of PubMed for articles published from January 2005 to December 2018, by use of the terms “Mtb,” “LTBI,” “diagnosis,” “biomarkers,” “prognosis,” “monitoring,” “transcriptomics,” and “proteomics.” Articles resulting from these searches and relevant references cited in those articles were reviewed. Articles published in English alone were included.

## Prospects of Detection of Biomarkers Associated With Mtb

### Recent Advances in the Detection of Mtb by Conventional Methods

It is widely accepted that the currently available diagnostic technology for TB detection is simply inadequate (Wallis et al., 2010). The most widely used diagnostic test to date is the microscopic detection of acid-fast bacilli (AFB) in sputum, which suffers from poor sensitivity ranging from 34–80% (Davies and Pai, 2008). This is because the AFB test requires at least 10,000 bacilli/ml of sputum to produce a positive result. If the concentration of bacilli falls below the cut-off, the chance to produce an AFB positive result is merely <10% (World Health Organization [WHO], 2004; Moro et al., 2010; Desikan, 2013). Sputum culture is relatively more sensitive than sputum AFB test but has a turnaround time of a few weeks. Furthermore, culture of Mtb requires Biosafety Level 3 facilities (World Health Organization [WHO], 2007), which are seldom available across TB endemic areas.

The recently developed PCR-based technique to amplify Mtb gene namely GeneXpert MTB/RIF represents a major breakthrough in TB diagnostics. The test is not only easy to operate, requiring less training for laboratory personnel, but is also capable of “killing two birds with one stone” by detecting Mtb and rifampicin-resistance simultaneously within 2 h (Zeka et al., 2011; Kwak et al., 2013), thereby significantly improving the rates of detection of Mtb. However, its use is only limited to active pulmonary TB (PTB), and not LTBI. Besides, all the sputum-based diagnostic methods have their own intrinsic limitations in that they are seldom useful in the detection of extra-pulmonary TB (EPTB) disease. Hence, the diagnosis of EPTB is reliant on sampling of site-specific tissues as well as other biological fluids such as pleural fluid and cerebral spinal fluid (CSF) which often involve invasive procedures (Goletti et al., 2016). This could be a real problem as the incidence of EPTB in some developing countries ranges from 13% to as high as 37% (Arora and Chopra, 2007; Gomes et al., 2014; Gaifer, 2017), and sampling often involves invasive procedures. Therefore, the use of host biomarkers that reflect the pathological process or host immune responses to active TB, EPTB, and LTBI could be a better choice.

### Developments in the Detection of Mtb DNA

From the pathogen perspective, detection of Mtb product such as Mtb DNA has been widely used as a diagnostic tool. GeneXpert has been shown to detect Mtb in a wide variety of clinical specimens including blood, urine, and CSF with better sensitivity and specificity as compared to Mtb culture (Cannas et al., 2008; da Cruz et al., 2011; Maynard-Smith et al., 2014; Theron et al., 2014). Sputum culture conversion either using solid and liquid media at the 2nd month post-initiation of TB therapy has long been used to monitor the efficacy of TB treatment, although this method usually take weeks (World Health Organization [WHO], 2013). GeneXpert MTB/RIF offers rapid detection in this regard and has shown good sensitivity (97%) and correlation time to culture positivity, but suffers from poor specificity ranging from 49 to 72% (Marlowe et al., 2011; Friedrich et al., 2013). This is in part due to GeneXpert, which is a PCR-based technique that detects total DNA present in the sample regardless live or dead bacilli present in clinical samples. Nonetheless, others have shown that patients who are positive for Mtb in blood are at an increased risk for death (Feasey et al., 2013). By using digital PCR (dPCR), a theoretically ten-fold more sensitive technique than real-time quantitative PCR, Li et al. developed a MTB detection test based on the MTB insertional sequence IS6110. This novel assay has been shown to have ∼twofold higher sensitivity than GeneXpert MTB/RIF assay in detecting MTB among probable and possible TB meningitis patients (Li et al., 2019).

### Prospects of Detecting Miscellaneous Components of Mtb

Other Mtb components such as the 17.5 kDa Mtb cell wall lipoarabinomannan (LAM) has also been used to detect the presence of Mtb in urine. However, all the commercial tests available thus far have low sensitivity. In a meta-analysis, Minion et al. (2011) showed that the sensitivity of urine LAM test in seven studies is highly variable ranging from 13 to 93%. Hamasur et al. (2015) had further improved the assay by concentrating the LAM antigen present in urine up to 5–100 times using immunoprecipitation method. This action has enhanced the sensitivity of the urine LAM assay, but only restricted to HIV-TB co-infected patients. The sensitivity of the assay among HIV-negative TB patients remains very low (Hamasur et al., 2015). Several possible reasons might explain the higher sensitivity of LAM assay in HIV patients; (i) there might be a higher Mtb load among HIV patients since they are immunodeficient (Boehme et al., 2005; Shah et al., 2010); or (ii) there might have been HIV-associated nephropathy among these patients that increases the glomerular permeability resulting in higher levels of LAM in urine (Doublier et al., 2007; Peter et al., 2010). Nonetheless, the assay has been used to monitor anti-TB therapy responses (Drain et al., 2015) and has also been shown to predict the onset of TB-associated immune reconstitution inflammatory syndrome (TB-IRIS) (Conesa-Botella et al., 2011) and death (Gupta-Wright et al., 2016) among TB-HIV co-infected patients.

Another Mtb secretory protein, the 30–35 kDa antigen 85 complex (Ag85A, Ag85B, and Ag85c) (Kashyap et al., 2005, 2007), early secretory antigen target-6 (ESAT-6), culture filtrate protein-10 (CFP-10) (Kalra et al., 2010; Feng et al., 2011; Shen et al., 2011; Zhang et al., 2015) and MPT64 (Kumar et al., 2011; Martin et al., 2011; Arora et al., 2015) have also been evaluated for their suitability as diagnostic reagents. The Ag85 complex is present in the sputum of patients with PTB (Kashyap et al., 2007) as well as in the CSF of patients with TB meningitis (Kashyap et al., 2005); but the sensitivity is inconsistent in various studies (Bentley-Hibbert et al., 1999; Kashyap et al., 2007). Other secretory proteins such as ESAT-6, CFP-10, and MPT64 are facing a similar problem. In one study, Turbawaty et al. attempted to detect the presence of all the three antigens in urine using a cocktail of polyclonal antibodies against all the three antigens. The authors showed that this strategy increased the sensitivity to 90%, although the specificity remained poor at <30% (Turbawaty et al., 2017).

## Prospects With Detection of Host Immune Biomarkers in Mtb Infection

### Host Antibody Responses to Mtb Antigens

Infection and immunity are the two sides of the same coin. When an individual is infected with Mtb, the pathogen will inevitably activate the immune response of the host leading to changes in host biomarkers. Pathogen-specific antibodies are the commonly used host biomarkers for pathogen diagnostics as they are simple to perform, inexpensive and are feasible for point-of-care. Many of the available serological tests employ either the lateral-flow or the ELISA format. A number of Mtb antigen-specific antibodies against PPD, antigen 60, ESAT-6, CFP-10, lipid-derived antigens and heat shock protein have been studied extensively (Verma and Jain, 2007). Of note, several Mtb antigens such as ESAT-6 and CFP-10 are not present in the genome of BCG strain, and hence detection of an immune response specific to these antigens can distinguish between and Mtb infection from vaccination responses (Andersen et al., 2000; Arend et al., 2000). Unfortunately, these assays so far have displayed poor sensitivity (ranges from 14% – 85%) and specificity (53–98.7%) (Steingart et al., 2007, 2009, 2011; Achkar and Ziegenbalg, 2012; Lagrange et al., 2014).

More recently, several highly antigenic MTB antigens have been developed for diagnostics with improved sensitivity and specificity than the classical ESAT-6- and CFP-10-based assays such as RV0310c-E and RV1255c-E. Receiver operating characteristic (ROC) analyses have shown that serum IgG against both RV0310c-E and RV1255c-E antigens has better sensitivity and specificity (AUC = 0.8 and 0.808, respectively) in diagnosing MTB compared to ESAT-6 and CFP-10 (AUC = 0.665 and 0.623, respectively) (Luo et al., 2017). Lopez-Ramos et al. (2018) showed that the antibodies against MTB antigen P12037 has a sensitivity and specificity of 92% and 91%, respectively in diagnosing active TB when used in concert with sCD14. Other researchers have found that antibody to MTB antigens such as proline-proline-glutamic acid protein 17 (PPE17) (Abraham et al., 2018) and mycobacterial DNA binding protein (MDP-1) (Maekura et al., 2019) can differentiate between patients with LTBI and active TB. Maekura et al. (2019) further showed that MDBP-1 may also be a good monitoring tool as persistently elevated IgG against MDBP-1 post anti-TB therapy could be associated with relapse after completion of treatment. Nonetheless, studies have also found that antibody responses against Mtb antigens are generally low among children (Achkar and Ziegenbalg, 2012) therefore, the use of serological tests for TB diagnosis, prognosis, and treatment monitoring can only be effectively used among adult patients.

One interesting study by Lu et al. on “resister,” a group of individuals highly exposed to MTB but who tested negative by T-cell based interferon gamma releasing assay (IGRA) and tuberculin skin test (TST), as well as did not develop LTBI has shed some light on TB pathogenesis. The authors found that the “resister” possessing MTB-specific IgM and class switched IgG; however, displayed reduced CD4-mediated IFN-γ responses toward ESAT-6, CFP-10, Ag85A, and Ag85B. Lu et al. (2019) also showed that the IgG of “resister” has higher avidity to MTB antigens compared to LTBI and healthy controls and further analysis also showed that the “resister” had significantly higher levels of IgG1 compared to other IgG subclasses. This suggests that the level of IgG1 could potentially be a prognosis biomarker, and holds the key to development of novel MTB therapeutics.

### Host Cytokine Responses to Mtb Antigens – Beyond Interferon-γ

Unlike antibody responses, the cellular immune responses against Mtb-specific antigens have shown better consistency. In the past, the TST has been widely used to diagnose active TB and LTBI. However, due to cross-reactivity, the test cannot differentiate between Mtb and other non-tuberculous mycobacterial infections as well as BCG vaccination. Besides, the TST also suffers from poor sensitivity among immunocompromised patients (Nahid et al., 2006; Frahm et al., 2011). Since the last decade, the T-cell based IGRA has emerged as the most popular tool in TB diagnostics (Ferrara et al., 2009). IGRA measure IFN-γ production after *ex vivo* stimulation of whole blood with Mtb-specific antigens such as ESAT-6 and CFP-10 (Lalvani et al., 2001b; Mori et al., 2004). There are two formats of IGRA assay, the ELISA-based QuantiFERON TB Gold assay (Mori et al., 2004) and the ELISPOT-based T-SPOT assay (Lalvani et al., 2001a). The T-SPOT assay has a higher sensitivity compared to QuantiFERON TB Gold assay in detecting active TB (91 and 80.2%, respectively) (Bae et al., 2016). In general, IGRA is sensitive and more specific than TST (Pai et al., 2008). Although IGRA is less affected by the HIV-status as compared to TST (Mendelson, 2007; Rangaka et al., 2007), the assay appears to perform poorly in children with advanced HIV infection (Hormi et al., 2018); however, IGRA performed using peripheral blood mononucleocytes (PBMC) isolated from specific sites of TB disease such as pleural fluid, bronchioalveolar lavage (BAL) and CSF has been found to be highly sensitive and specific (Losi et al., 2007; Thomas et al., 2008).

Some studies suggested that IGRA response is stronger in active TB than LTBI, and hence, allows the differentiation of the two forms of TB disease (Janssens, 2007). However, other studies suggested that IGRA may not be suitable for the diagnosis of active TB and LTBI in high TB-burden regions (Sharma et al., 2017). This may be attributed to the nature of the antigen used in the IGRA, i.e., ESAT-6 and CFP-10 as they are secretory proteins of MTB especially during active infection. One study by Arroyo et al. (2018) showed that the use of latency-related antigens, i.e., dormancy survival regulon (DosR) and resuscitation promoting factor (Rpf) in IGRA could be better. The DosR peptide RV2029c and the Rpf peptide RV2389c have shown to differentiate LTBI from active PTB with a sensitivity of 90 and 85%, respectively (Arroyo et al., 2018).

Given the sensitivity and specificity of IGRA, the assay has also been suggested for use as a treatment monitoring tool (Ribeiro et al., 2009; Bocchino et al., 2010; Chee et al., 2010). Several studies have shown that patients who are IGRA negative on completion of anti-TB therapy experienced complete clinical and microbiological recovery (Goletti et al., 2008; Kabeer et al., 2011; Helmy et al., 2012). Another cohort by Kaneko et al. (2015) showed that patients who were IGRA positive at the end of treatment developed TB reactivation; whilst those who were IGRA negative did not develop TB reactivation for 2 years of follow-up.

One advantage of IGRA as an *ex vivo* stimulation assay is that the same assay tubes can be re-used to study other biomarkers either by multiplex cytokine bead array or by flow cytometry to obtain “biosignature” that may differentiate between different stage of TB disease (Chegou et al., 2009). One biomarker at the downstream of IFN-γ, i.e., IP-10 has shown to be of good use as a biomarker. Elevation of plasma level of IP-10 in un-stimulated tubes has been associated with active TB (Azzurri et al., 2005; Whittaker et al., 2008; Lighter et al., 2009; Novel et al., 2013; Petrone et al., 2015). IP-10 not only is as sensitive as IFN-γ (Kabeer et al., 2011) in blood but also offer several additional advantages. For instance, detection of IP-10 in the urine of children (Petrone et al., 2015) and adults (Darrah et al., 2007) with active TB makes sample collection easier. Further, unlike IFN-γ, IP-10 is less affected by HIV status; making it a robust biomarker to be used in *ex vivo* stimulation assays (Ruhwald et al., 2008; Goletti et al., 2010a, b; Kabeer et al., 2011).

Several studies have been conducted using multiplex cytokine bead array on plasma with or without *ex vivo* stimulation to differentiate active TB and LTBI. These studies employ a combination of 5–15 biomarkers in their analysis whose sensitivity ranges from 82.3 to 96.7% (Mihret et al., 2013; Won et al., 2017; La Manna et al., 2018). Despite the combination of biomarkers used by different studies, IFN-γ and IP-10 are the most common biomarkers used in these studies. Besides, other studies have shown that the ratio of IFN-γ and IL-10 (Sai Priya et al., 2010), ratio of IL-2 and IFN-γ (Wu et al., 2017), IL-8, IP-10, MIP-1α, sIL-2Rα, vascular endothelial growth factor (VEGF), MCP-3 (Yao et al., 2017; Hoel et al., 2019) as well as soluble markers to TLR-4 pathway such i.e., sCD14, MD-2, and LPS (Feruglio et al., 2013) can distinguish between active TB and LTBI and can also correlate with treatment success. By screening 38 cytokines, Luo et al. (2019) found that by using a combination of three cytokines, i.e., eotaxin, CCL22 and MCP-1, they were able to discriminate LTBI from active TB with a sensitivity and specificity of 87.8 and 91.8%, respectively (AUC = 0.94). Other plasma markers that associated with treatment success (measured as time to sputum conversion) include IL-6, MCP-1 (Djoba Siawaya et al., 2009), VEGF (Riou et al., 2012), hemeoxygenase-1 (HO-1), matrix metalloproteinases (MMP) (Andrade et al., 2013), serum amyloid, proteasome activation complex subunit-1, IL-11 receptor antagonist, and 2-antiplasmin (Nahid et al., 2014). However, further studies are required to evaluate and validate these markers.

### Host Cellular Immune Responses to Mtb Antigens

Flow cytometry is a powerful technique used to analyze the characteristics of individual cells within heterogeneous populations. In principle, following Mtb antigen exposure, CD4^+^ and CD8^+^ T cells undergo several stages of differentiation from naïve T-cells (T_N_) progressing to central memory (T_CM_), effector memory (T_EM_) and finally to terminally differentiated T cells (T_EMRA_) (Harari et al., 2011; Rovina et al., 2013); and the more antigenic exposure (in quantity, antigenicity, and duration) of T cells the more advance the cellular differentiation. Based on this principle, several efforts have been made to characterize the functional signature of T cells (i.e., the combination of subsets and their cytokine production) that associated with stages of TB disease. CD69 is a co-stimulatory receptor and an early activation marker (Borrego et al., 1999; Yong et al., 2017) and increased levels of CD4^+^CD69^+^IFN-γ^+^ T cells is associated with early active TB or recent TB infection (Nikolova et al., 2013). Similarly, the frequency of CD137, a co-stimulatory molecule responsible to sustain effective activation, proliferation and survival of T-cells has also been shown to be associated with active TB (Yan et al., 2017).

A study done by Millington et al. (2007) reported that the polyfunctional CD4^+^ IFN-γ^+^IL-2^+^TNF-α^+^ T cells are predominantly seen in patients with active TB as compared to CD4^+^IL-2^+^IFN-γ^+^ T cells and CD4^+^IFN-γ^+^ only in LTBI. More detail studies found that the non-active form of TB response including LTBI or BCG vaccination and treated TB are associated with predominant CD4^+^IFN-γ^+^IL-2^+^ T_EM_ and CD4^+^IL-2^+^ T_CM_; whilst active TB is associated with predominantly CD4^+^IFN-γ^+^ T_EMRA_ cells (Sutherland et al., 2009; Caccamo et al., 2010; Casey et al., 2010; Sester et al., 2011). By using CD38, an immune activation marker and CD27, a maturation marker, Ahmed et al. (2018) showed that active TB was associated with increased frequency of CD38 + CD27^low^; whilst LTBI was associated with CD38-CD27^high^. Another study showed that LTBI is associated mostly with polyfunctional CD4^+^ T cells expressing IFN-γ, IL-2, and TNF-α and in combination; whilst active TB is predominated with CD4^+^ T cells expressing only TNF-α, and not IFN-γ as measured by IGRA (Harari et al., 2011). CD27, a member of TNF-α receptor superfamily was found to be useful in differentiating active TB and LTBI. Streitz et al. (2007) showed that active TB patients had significantly higher CD4^+^CD27^+^ T cells as compared to BCG vaccinees and patients with LTBI had an intermediate level of CD4^+^CD27^+^ T-cells. Other studies found that CD27 (Adekambi et al., 2012; Nikitina et al., 2012; Petruccioli et al., 2013, 2015; Portevin et al., 2014) and Mtb-specific CD4^+^ T cells (Adekambi et al., 2012; Nikolova et al., 2013) correlate with Mtb antigen/bacilli burden and hence might serve as good biomarkers for monitoring treatment progress. Other subpopulations of T cells such as IL-10 + Th17 T cells were found to be significantly higher among LTBI; whilst IFN-γ + Th17 was significantly higher in active TB when stimulated with DosR (Rakshit et al., 2017). Besides T-cells, dendritic cells, especially the percentage of BDCA3 + mDC and CD123 + pDCs were significantly reduced in patients with active TB; while the same subtypes were found to be significantly activated among patients with LTBI (Parlato et al., 2018).

Several other surface markers including the immune activation marker CD38 and HLA-DR, the proliferation marker Ki-67 (Adekambi et al., 2012) as well as the percentage of myeloid-derived suppressor cells (MDSCs) (El Daker et al., 2015) have also been suggested as biomarkers to monitor treatment efficacy. The expression of immune activation markers such as CD38 and HLA-DR on T cells was significantly reduced by week 9 after initiation of the anti-TB therapy. The slope of decline in the expression of these markers was correlated with the time of stable culture conversion (Ahmed et al., 2018). Study also showed that individuals had a substantial amount of T_EM_ at the sixth month of anti-TB therapy suggesting that persistence of live Mtb may lead to relapse; while individuals who retained only T_CM_ may hint complete clearance of Mtb (Goletti et al., 2006; Butera et al., 2009; Millington et al., 2010; Wang et al., 2010; Pollock et al., 2013; Chiacchio et al., 2014; Petruccioli et al., 2015). Similarly, follow up on the anti-TB treatment showed that patients who had significant reduction in TB load showed a shift from CD4^+^IFN-γ^+^ T_EMRA_ cells to CD4^+^IFN-γ^+^IL-2^+^ T_EM_ (Caccamo et al., 2010). In a longitudinal prospective study on active TB on anti-TB therapy, Ferrian et al. studied the association between Tregs and treatment efficacy. By using cut-off point at day 71 after initiation of anti-TB therapy, the authors classified the patients into two groups: (i) those who achieved stable sputum culture conversion faster than day 71 as rapid responders, and (ii) those achieved stable sputum culture conversion later than day 71 as slow responders. The authors found that the frequencies of Treg was significantly higher in slow responders and could predict time to stable culture conversion with the sensitivity of 81% and specificity of 85% (AUC = 0.87).

## Prospects of Biomarkers by Unbiased “Omics” Approach

Omics approach, i.e., genomic, transcriptomic, proteomic and metabolomics is a high throughput method that allows thousands of biomarkers of multi-dimension, to be unbiasedly acquired in one step (Kell and Oliver, 2004). While genomics provide an overview of genetic instruction provide by DNA, transcriptomics would investigate the gene expression patterns, proteomics the dynamic of protein products, and metabolomics the interactions and understanding of the entire metabolism of an individual in a disease setting. These “omics” approaches have been used not only in TB diagnosis, monitoring treatment efficacy, predicting treatment outcomes, but also used to improve understanding the pathogenesis of TB disease.

One genomic study has investigated SP110, a gene encoded for IFN-induced nuclear protein in a large cohort of patients including 301 active TB, 68 LTBI and 278 healthy controls. From the 5 index SNPs, i.e., rs7580900, r7580912, rd9061, rs11556887, and rs2241525, the study identified that rs9061 was significantly associated with increased susceptibility to LTBI (Chang et al., 2018). Further investigations indicated that individuals bearing this SNP had decreased levels of plasma TNF-α (Leu et al., 2018).

One transcriptomic study identified a profile of 393-transcripts signatures in whole-blood characterizing active TB; and 86-transcript signature that distinguishes TB from other infections. Through modular and pathway enrichment analysis the study revealed that active TB was predominated with neutrophil-driven interferon-inducible genes, consisting of both IFN-γ and type I IFN-αβ signaling (Berry et al., 2010). These findings have been further validated by several independent cohorts (Lesho et al., 2011; Maertzdorf et al., 2011a, b; Ottenhoff et al., 2012; Bloom et al., 2013; Kaforou et al., 2013; Walter et al., 2015) and the profile of transcript signatures decreased after the initiation of treatment (Ottenhoff, 2009; Bloom et al., 2013; Cliff et al., 2016). Moreover, Anderson et al. (2014) also showed that the transcription profile was able to distinguish between active TB and LTBI.

The cytotoxic cell gene transcripts may also be used for end treatment assessment to predict TB relapse (Joosten et al., 2012; Cliff et al., 2016). Maertzdorf et al. (2011b) found that the high-affinity IgG Fc receptor IB (FcγRIB) along with other four different transcripts are differentially expressed among between active TB and LTBI. Other transcripts such as lactotransferrin, CD64 (also known as FcγR1A) also can discriminate active TB from LTBI (Sutherland et al., 2014). Another study by Lee et al. (2016) found that genes related to innate immune responses are highly expressed among patients with active TB; whilst genes related to apoptosis and natural killer (NK) cell activation are predominantly expressed in patients with LTBI. RAS and RAB interactor 3 (RIN3) also could discriminate between active TB, LTBI, and recurrent infection (Mistry et al., 2007).

In the pass, the transcriptomic studies have mainly studied the profiling of mRNA expression, but more recently, there has been a growing interest on the non-coding region of mRNA. Although these non-coding RNA does not encode for any protein, they do possess certain regulatory functions and hence may be altered by different stages of TB disease. By comparing the micro RNA (miRNA) profile of children infected with TB, adult patients with active PTB, active EPTB, TB/HIV co-infection as well as LTBI, Miotto et al. (2013) managed to identify a set of 15 miRNA signature that was common for TB infection with a sensitivity and specificity of 82 and 77%, respectively. Another study by Fu et al. has looked into the circular RNA (circRNA) and their association with TB disease. circRNA is a recently discovered, endogenous, covalently closed without free 3′- and 5′-end non-coding RNA. Being a covalently closed circular RNA, it is highly resistant to RNase degradation and hence are abundant and long-lasting in cells. The authors found that there were 171 deregulated circRNA in TB infection where circRNA_103017, circRNA_059914, circRNA_101128 were most profoundly elevated whilst circRNA_062400 was decreased. This circRNA signature could potentially be a useful marker for TB (Fu et al., 2019). Chakrabarty et al. (2019) also identified several miRNA including 2 from human, i.e., hsa-miR-146a-5p and hsa-miR-125b-5p and one miRNA from MTB, i.e., MTB-miR5 that increased among patients with active TB. miRNA has also been used to differentiate LTBI from active TB. By studying 250 miRNAs, Lyu et al. (2019) also identified the patterns where the hsa-let-7e-5p, hsa-let-7d-5p, hsa-miR-450a-5p, and has-miR-140-5p were differentially expressed among patients with LTBI; whilst hsa-miR-1246, hsa-miR-2110, hsa-miR-370-3p, hsa-miR-28-3p, hsa-miR-193b-5p were associated differentially expressed among patients with active TB.

Besides, by using proteomic microarray method, Hai et al. screened 4262 MTB antigens and found that IgG toward 152 Mtb antigens were differentially elevated among patients with active TB when compared to patients with LTBI. Further analysis showed that RV2031c, RV1408 and RV2421c were able to discriminate between active TB and LTBI (Cao et al., 2018). By studying 1011 host serum biomarkers, Liu et al. found that 153 protein were significantly elevated among patients with severe TB. These included α-1-acid glycoprotein 2 (ORM2), IL-36α, s100 calcium-binding protein (S100-A9), and superoxide dismutase (SOD) (Liu et al., 2018). Aiming to improve understanding on TB progression, Duffy et al. investigated a cohort of household contacts of TB index cases HHCs and non-human primate challenge model. By combining both blood transcriptomic, serum metabolomics and pathway analysis, the authors identified a novel immunemetabolic signature involving cortisol, tryptophan, glutathione and tRNA acylation that associated with the progression of latent to active TB (Duffy et al., 2019).

Summary of each biomarkers and their applications are given in Table 1.

**TABLE 1:** Biomarkers for diagnosis, prognosis, and monitoring of MTB infection.

	**Biomarker**	**Specimen**					**Application**					**Remark**
				
		**Sputum**	**Body fluids**	**CSF**	**Blood/serum/plasma**	**PBMC**	**Mtb diagnosis**	**Distinguish active TB vs. LTBI**	**Monitoring**	**Predict relapse/worsen/progress**	**Predict treatment success**	
Microbiology technique	AFB staining	∙	∙	∙	∙		∙		∙			– Rapid, convenient and inexpensive test
												– Non-specific, must accompanied with confirmation tests
												– Limited sensitivity; required at least 5000 AFB/mL to be detected
												– High false negative rate
	Mtb culture	∙	∙	∙	∙		∙		∙			– Long turnaround time (3–8 weeks)
												– Required biosafety level three facilities to handle Mtb culture

Detection of Mtb components	Mtb DNA detection (GeneEpert)	∙	∙	∙	∙		∙			∙		– Rapid, diagnosis, and detection of drug resistant Mtb
												– Low sensitivity (49–72%)
												– Patient positive with Mtb in blood assoc. with increased risk of death
	Mtb antigens ∙ LAM		∙						∙	∙		– Low sensitivity (13–93%)
												– Use to monitor anti-TB response in TB-HIV
												– co-infected patients
												– Predict TB-IRIS and death among TB-HIV co-infected patients
	Mtb antigens ∙ Ag 85 complex, ESAT-6, CFP-10, MPT64	∙	∙	∙	∙							– Sensitivity is in consistent Poor specificity
	Digital PCR (dPCR)				∙		∙					– Supreme sensitivity then conventional qPCR
												– Twofold higher sensitivity than GeneXpert in detecting MTB among probable/possible TB meningitis
												– The study uses CSF, but can be apply for sputum, serum/plasma and other body fluid
Host antibodies responses against ex-vivo stimulation of Mtb Ags	PPD, Ag60, ESAT-6, CFP-10	∙			∙							– Poor sensitivity (14–85%); poor specificity (53–98%)
												– Antibody response usually very low among children
	RV0310c-E				∙		∙					– Better sensitivity than ESAT-6 and CFP-10
	RV1255c-E											
	P12037				∙		∙					– Sensitivity = 92%, specificity = 91%
	PPE17				∙		∙	∙	∙			– More antigenic antigen than ESAt-6 and CFP-10
	MDP-1											
	RV2031c, RV1408, RV2421c				∙		∙	∙				– IgG against these three Ags were initial identified by screening done by proteomics

Host cytokines responses against ex-vivo stimulation of MTB Ags	Tuberculin skin test (TST)	–	–	–	–	–	∙	∙	∙			– Poor sensitivity among HIV/immunocompromised patients
	**Ag:** ESAT-6, CFP-10 IFN-γ (IGRA)				∙	∙	∙			∙	∙	– T-SPOT sensitivity (91.2%); QuantiFERON sensitivity (80.2%)
												– More specific than TST
												– Less affected by HIV-status compared to TST
												– Predict TB-reactivation within 2 years
												– Associated with complete clinical and microbiological recovery
	**Ag:** ESAT-6, CFP-10 IP-10				∙		∙	∙				– High IP-10 in unstimulated tube associated with active TB
												– Less affected by HIV status
	**Ag:** ESAT-6, CFP-10 sCD14, MD-2, LPS				∙		∙	∙			∙	– Distinguish between active-TB and LTBI
												– Levels correlated with treatment success
	**Ag:** ESAT-6, CFP-10 IL-8, MIP-1a, sIL-2Ra, VEGF, MCP-3				∙		∙	∙				
	**Ag:** ESAT-6, CFP-10 IL-6, MCP-1, VEGF, HO-1, MMP, IL-11R antagonist, 2-antiplasmin				∙						∙	
Host cytokines responses against ex-vivo stimulation of MTB Ags	**Ag:** ESAT-6, CFP-10 ratio of IL-2/IFN-γ				∙		∙	∙				
	**Ag:** ESAT-6, CFP-10 eotaxin, CCL22, MCP-1				∙		∙	∙				– When used in combination, the sensitivity = 87.8% and specificity = 91.8%
	**Ag:** DosR, RV2029c, Rpf, RV2389c IFN-γ (IGRA)				∙	∙	∙	∙				– Both DosR and Rpf are antigen expressed during latent infection
												– When used in combination, the sensitivity = 90% and specificity = 85%

Host cellular immune responses against ex-vivo stimulation of Mtb antigens	CD4 + CD69 + IFN-γ+					∙	∙	∙				– Associate with early or recent Tb-infection
	CD4 + IFN-γ + IL-2 + T_EM_					∙	∙	∙				– Associated with LTBI
	CD4 + IL-2 + T_CM_					∙	∙	∙				– Associated with LTBI
	CD4 + IFN-γ + T_EMRA_					∙	∙	∙			∙	– Associated with active TB-infection
												– Shift of functional signature from CD4 + IFN-γ + T_EMRA_ to CD4 + IFN-γ + IL-2 + T_EM_ after completion of ATT indicate successful treatment
	CD4 + IFN-γ + IL-2 + TNF-α+					∙	∙	∙			∙	– Associated with active TB-infection
	CD4 + IFN-γ + IL-2+					∙	∙	∙			∙	– Associated with active LTBI
	CD4 + IFN-γ+					∙	∙	∙			∙	– Associated with active LTBI
												– Shift of functional signature from CD4 + IFN-γ + TNF-α + to CD4 + IFN-γ + IL-2 + or CD4 + IFN-γ + after completion of ATT indicate successful treatment
	T_EM_ T_CM_					∙				∙	∙	– High T_EM_ at sixth months of ATT assoc. with TB reactivation
												– High T_CM_ at sixth months of ATT assoc. with complete clearance of TB
	CD4 + CD27+					∙	∙	∙				– Differentiate between active TB and LTBI
												– High CD4 + CD27 + associated with active TB
												– Intermediate CD4 + CD27+ associated with LTBI
	CD137 + T-cells				∙	∙						– Is a member of TNF receptor superfamily
												Associated with active TB
	IL-10 + Th17				∙	∙		∙				– Associated with LTBI, when stimulated with DosR
	IFN-γ + Th17				∙	∙		∙				– Associated with active TB, when stimulated with DosR
	%BDCA3 + mDC				∙	∙		∙				– Reduction in% indicated active TB infection
	%CD123 + pDC											
	MFI BDCA3 + mDC				∙	∙		∙				– Increase activation markers in these subsets indicated LTBI
	MFI CD123 + pDC											
	CD38, HLA-DR								∙	∙	∙	– Used for monitoring of time to culture conversion after initiation of anti-TB therapy
												– Slope of reduction in CD38 and HLA-DR correlated with time to culture conversion
	Treg								∙	∙	∙	– Low% of Treg found among rapid responder
												– Percentage of Treg inversely correlated with time to culture conversion

Genomics, transcriptomic, proteomics, and metabolomics	∙ Neutrophil derived IFN-γ, IFN-α and β					∙	∙	∙				– Further validations required
	∙ FcγR1B					∙	∙	∙				– Further validations required
	∙ Lacto transferrin CD64, RIN3					∙	∙	∙				– Further validations required
	**circRNA** _103017, _059914, _101128, _062400				∙	∙		∙				– Covalently closed circular RNA, highly resistant to RNase, hence presence in abundance in cytoplasm
												– Increase in these three circRNAs is associated with LTBI
												– Decreased in this circRNA is associated with active TB infection
	**host miRNA**				∙		∙	∙				– Increase in these miRNA is associated with active TB infection
	_hsa-miR-146a-5p											
	_hsa-miR-125b-5p											
	**MTB miRNA**											
	_MTB-miR5				∙		∙	∙				
	**Host miRNA**				∙		∙	∙				– Elevation of these miRNA were associated with LTBI
	_hsa-let-7e-5p											
	_hsa-let-7d-5p											
	_hsa-miR-450a-5p											
	_hsa-miR-140-5p											
	**Host miRNA**				∙		∙	∙				– Elevation of these miRNA were
	_hsa-miR-1246											
	_ hsa-miR-2110											– associated with active TB infection
	_ hsa-miR-370-3p											
	_ hsa-miR-28-3p											
	_ hsa-miR-193b-5p											
	**Host proteomics**				∙					∙	∙	– Elevation of these plasma markers were associated with severe TB infection
	ORM2, IL-36α,											
	S1000-A9, SOD											
	**Host metabolomics** ∙ Cortisol, tryptophan, glutathione, tRNA acylation				∙					∙		– Predict progression from LTBI to active TB (applicable to host hold contact of TB infected individual)
	**Host genomics** ∙ SNP of SP110 gene (rs9061)					∙				∙		– SP100 gene encoding for IFN induced nuclear protein
												– Individual bearing this SNP was associated lower plasma level of TNF and increase susceptibility to LTBI

## Future Developments and Conclusion

As omics approach is capable to provide a more holistic picture for the disease mechanisms and hence more accurate in predicting disease outcomes (Clarke et al., 2008; Heidecker et al., 2008; Gesthalter et al., 2015; Jong et al., 2016; Kohonen et al., 2017; Lowe et al., 2017). Based on the omics profile, new hypotheses will be generated for further examination. There have already been a few successful cases in the search for TB biomarkers (Weiner et al., 2012; Kaforou et al., 2013; Goletti et al., 2016; Maertzdorf et al., 2014, 2016; Weiner and Kaufmann, 2017) and the number of study is still increasing. It is also worthwhile to point out that Mtb-specific immune responses are probably not homogenous in human populations and might be influenced by HIV-1 co-infection, heredity and several other exogenous environmental factors [183–185]. State-of-the-art data mining tools including supervised and unsupervised learning as well as new algorithms must be designed to handle such big data. Further, since the number of variables in omics studies is usually way larger than the sample size, the statistical power for detecting a few suitable biomarkers will inevitably decrease profoundly. Given that high investment is required for omics studies, which obviously may be impractical for developing countries, the well-validated omics markers should be applied for simple and rapid point-of-care tests.

## Author Contributions

YY and ES wrote the first draft of the manuscript. HT, AS, WW, RV, RE, VV, and ML contributed to the writing of the manuscript. YY, HT, AS, WW, VV, ML, and ES agreed with the manuscript’s results and conclusion. All authors have read and confirmed that they meet, ICMJE criteria for authorship.

## Conflict of Interest

The authors declare that the research was conducted in the absence of any commercial or financial relationships that could be construed as a potential conflict of interest.
